# Pulsed Radiofrequency Combined With Methylene Blue Paravertebral Nerve Block Effectively Treats Thoracic Postherpetic Neuralgia

**DOI:** 10.3389/fneur.2022.811298

**Published:** 2022-06-03

**Authors:** Meiduan Ji, Peng Yao, Zhenkai Han, Danlin Zhu

**Affiliations:** ^1^Department of Pain Management, Shengjing Hospital of China Medical University, Shenyang, China; ^2^Department of Anesthesiology, Women and Children's Hospital, School of Medicine, Xiamen University, Xiamen, China

**Keywords:** postherpetic neuralgia (PHN), pain management, pulsed radiofrequency (PRF), methylene blue (MB), paravertebral nerve block

## Abstract

**Objective:**

To compare the effect, safety, and patient satisfaction of pulsed radiofrequency combined with methylene blue paravertebral nerve block and pulsed radiofrequency alone in the treatment of thoracic postherpetic neuralgia (PHN).

**Methods:**

A total of seventy-two patients with PHN diagnosed in the Department of Pain Management of Shengjing Hospital at China Medical University, from September 2019 to April 2021, were enrolled in the study. Patients were randomly divided into two groups. Group A (*n* = 36) received pulsed radiofrequency treatment. Group B (*n* = 36) received pulsed radiofrequency + methylene blue paravertebral nerve block. Patients were followed-up at 1 day, 1 week, 1 month, 3 months, and 6 months after treatment. Observation at each follow-up included basic patient characteristics, Visual Analog Scale (VAS), Hospital Anxiety and Depression Scale (HAD), the Insomnia Severity Index (ISI), patient satisfaction, complications, and side effects.

**Results:**

Compared with preoperative values, the VAS scores significantly decreased in both groups at each postoperative time point (1 day, 1 week, and 1, 3, and 6 months; all *p* < 0.05). Compared with group A, VAS scores in group B were significantly lower 1 week and 1 month after surgery (*p* < 0.05). Patients in group B had lower HAD scores than those in group A 1 week after surgery (*p* < 0.05). Patients in group B had lower ISI scores than those in group A 1 day, 1 week, and 1, 3, and 6 months after surgery (*p* < 0.05). The pregabalin dosage in group B was lower than that in group A at 1 and 6 months after surgery (*p* < 0.05). Patient satisfaction was higher in group B than in group A at 1 week and 6 months after surgery (*p* < 0.05). There were no serious complications or side effects in either group.

**Conclusion:**

Pulsed radiofrequency combined with methylene blue paravertebral nerve block is superior to pulsed radiofrequency alone in the treatment of thoracic PHN, which can significantly relieve PHN and improve the condition of sleep and emotional disorders. Therefore, it is a safe and effective treatment method.

## Introduction

Postherpetic neuralgia (PHN) is a common disorder causing refractory neuropathic pain (1). The main manifestations (2) are severe pain in skin lesions that feel like needling, burning, or electric shock, which can be unbearable for patients. Patients with PHN suffer a decreased quality of life, can develop insomnia, and even anxiety and depression. This is not only a cause of distress for patients, but also prone to causing fatigue, stress, insomnia, and emotional distress for their families (3). At present, there are several clinical therapies for PHN, including analgesic drugs (4), nerve block (5), radiofrequency therapy (6), analgesic pump implantation (7), and spinal cord electrical stimulation (8). However, their therapeutic effects are still controversial. As the most common long-term complication of herpes zoster (HZ), PHN is difficult to treat and has a low cure rate. Furthermore, current evidence is insufficient to determine a single optimal therapy. In addition, the incidence of HZ increases with age. Because the population structure of many countries is gradually aging, the number of HZ and PHN patients may increase significantly in the future. This is bound to bring great clinical challenges. To address these challenges, we must further study effective treatment methods.

Pulsed radiofrequency (PRF) has been widely used in the treatment of neuropathic pain due to its reversibility, low nerve damage, high safety, and easy application (9). However, there are different opinions about the effectiveness of PRF therapy for PHN. Some reports believe that PRF can effectively relieve pain and improve the quality of life of patients with PHN (10–13). Other reports suggest that PRF is not the ideal therapy for treating neuropathic pain (14) and that PRF does not significantly relieve nerve root pain (15). Methylene blue (MB) can cause reversible damage to the nerve, reducing nerve sensitivity without damage to the nerve cell structure, therefore achieving an analgesic effect. It can maintain local anesthesia for about 20 days. A nerve block can be used to relieve long-term pain or eliminate permanent pain, especially for refractory neuropathic pain (16, 17). Some studies report that PRF and nerve block effectively treat PHN (18). Other studies have used MB alone for nerve block, but PRF combined with MB paravertebral nerve block for thoracic PHN has not been reported; thus, this study intended to observe the efficacy and safety of PRF combined with MB paravertebral nerve block in the treatment of thoracic PHN to guide clinical application.

## Materials and Methods

### Patients

**Figure 1:**
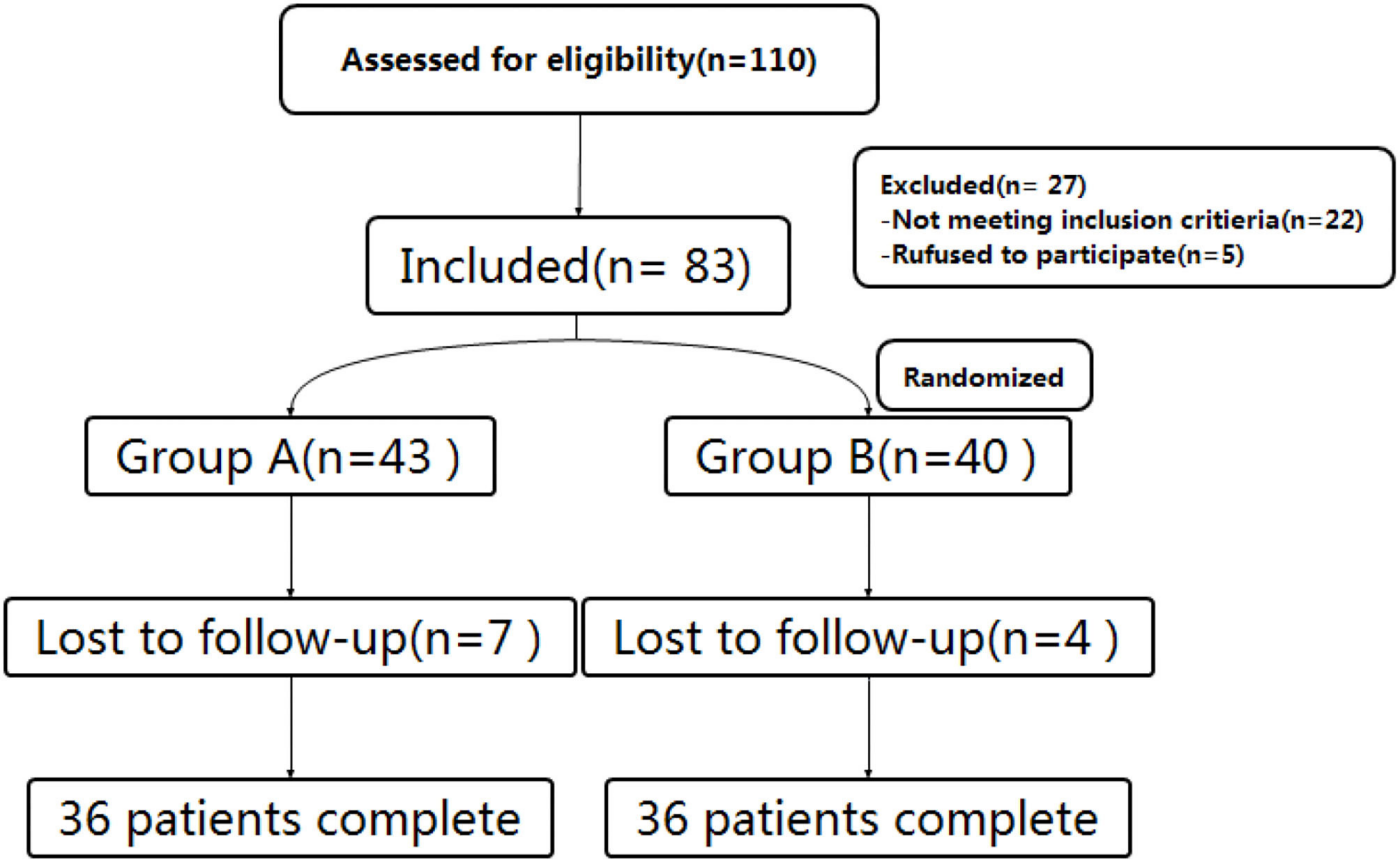
Study flowchart. All 72 patients were included in the treatment.

From September 2019 to April 2021, patients with PHN involving thoracic nerves who met the inclusion criteria were enrolled in this study at the Department of Pain Management of Shengjing Hospital of China Medical University. All patients enrolled had a course of disease ≥ 3 months. Based on our clinical experiences, most patients with PHN are over 50 years old. Thus, to reduce age-induced statistical errors, we chose to exclude patients who were younger than 50 years. All patients were treated with pregabalin (300–450 mg/day according to the patient's condition) with a poor response (VAS ≥ 5). Before surgery, patients were informed of the risks and complications and signed informed consent. The study was approved by the Ethics Committee of Shengjing Hospital, China Medical University (ID: 2017SP134K). As per a random number table, patients were randomly divided into two surgical groups (Figure 1).

### The Inclusion Criteria Were as Follows

① Patients who met the diagnostic criteria of PHN (19), that is, intense neuropathic pain that lasts for more than 90 days after the acute rash is cured
② Patients with PHN involving the thoracic nerve
③ Age > 50 years
④ Patients with poor pain control after drug treatment (VAS ≥ 5)
⑤ Patients were graded as level I-II according to the American Society of Anesthesiologists (ASA) classification.

### The Exclusion Criteria Were as Follows

① Infections
② Abnormal coagulation function
③ Severe cardiopulmonary, liver, or kidney insufficiency
④ Thoracic space-occupying lesions, including syringomyelia and other spinal canal space-occupying entities
⑤ Mental illness.

### Surgical Procedure

**Figure 2:**
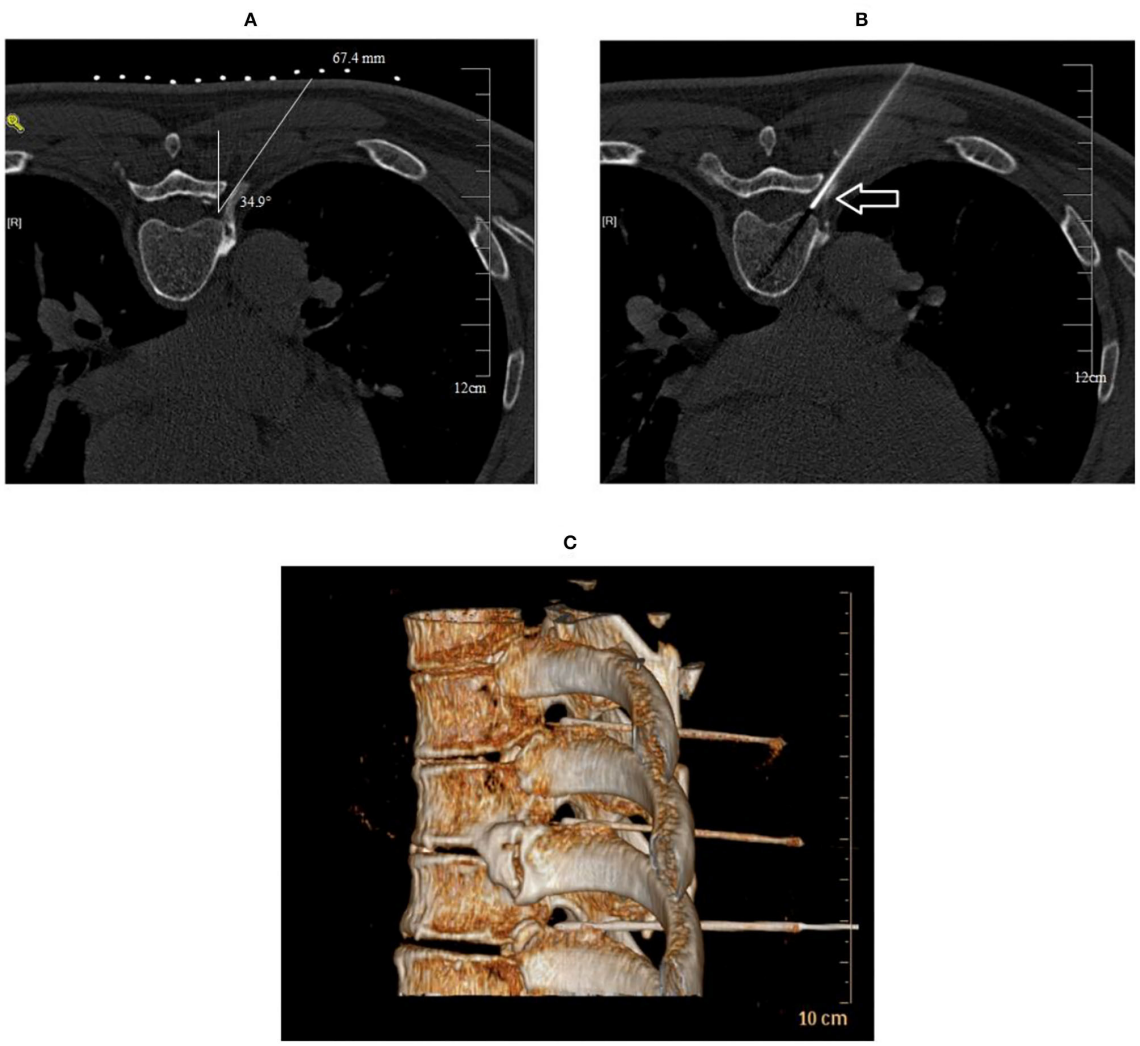
CT-guided puncture route and drug administration. **(A)** A CT plain scan shows how under CT guidance, a safe route was chosen to avoid injury to the vessel, pleura, and lung. The puncture point and puncture angle were clearly defined by a computer. **(B)** A CT plain scan shows that the radiofrequency needle was located at the spinal nerve root on the left side and adjacent to the pleura and lung as indicated by the arrow. **(C)** A 3D CT reconstruction shows the puncture needles of the three segments of the thoracic vertebrae were located at the intervertebral foramen as indicated by the arrow.

1) **Preoperative**: After admission, the patients' involved ganglion segments were identified based on self-reported areas of pain on the body surface and electromyogram examination findings.
2) **Surgical process**: With vital signs being monitored, patients were placed in the prone position and computed tomography (CT) was used to confirm the puncture route. After local anesthesia with 0.5% lidocaine, a radiofrequency puncture needle (20 Ga, 145 mm, 5 mm) was inserted at the CT positioning angle and gradually advanced under CT guidance until the tip touched the dorsal root ganglion of the targeted nerve in the intervertebral foramen (Figure 2). Then, a 50 Hz, < 0.2 V sensory test was performed to induce numbness in the innervated area of the nerve. Subsequently, a 2 Hz, < 0.5 V motor test was performed to ensure there were no tremors over the innervated area. The puncture was successful if there was no muscle tremor in the dominant area at < 0.5 V, and the PRF therapy could be performed. The PRF apparatus parameters were as follows: 42°C for 600 s (pulse width 20 ms and frequency 2 Hz). After PRF treatment, 0.5 ml of betamethasone (5 mg + normal saline 1 ml) was given to each dorsal root ganglion. Finally, patients in group A were given 0.5 ml of normal saline, and patients in group B were given 0.5 ml (2 mg) 0.2% MB. Each involved ganglion segment was treated sequentially depending on the number of involved ganglion segments of patients. After treatment, the needle was withdrawn, and the puncture site was covered with a dressing. Patients were returned to the ward by a flat cart, and their vital signs were dynamically monitored.

### Observations and Follow-Up

Preoperative general information was recorded, including sex, age, weight, height, body mass index (BMI), pain duration, VAS, ISI, HAD, and daily dosage of pregabalin. VAS, ISI, HAD, patient satisfaction, and daily dosage of pregabalin were recorded at 1 day, 1 week, 1 month, 3 months, and 6 months after surgery by pain physicians who were not involved in the operation. The whole process strictly followed the double-blind principle.

#### Visual Analog Scale

The VAS was used to evaluate pain, 0 indicates no pain and 10 indicates unbearable severe pain.

#### Hospital Anxiety and Depression Scale

The HAD (20) is a self-rating scale widely used in clinical diagnosis and general hospitals to assess anxiety and depression. The HAD scale consists of 14 items, which are divided into two subscales of anxiety and depression. The anxiety scale and depression scale each contain seven items, and each item is rated on a scale of 0–3. The total score for anxiety and depression range from 0 to 21. A HAD scale score > 7 is used to distinguish anxiety and/or depression, 8–10 is classified as mild anxiety and/or depression, 11–14 is classified as moderate anxiety and/or depression, and 15–21 is classified as severe anxiety and/or depression.

#### The Insomnia Severity Index

The ISI (21) is an effective 7-item self-report scale that can assess the severity of insomnia in the past 2 weeks. The total score (0–28) was calculated, with 0–7 as clinically insignificant insomnia, 8–14 as subclinical insomnia, 15–21 as moderate clinical insomnia, and 22–28 as severe clinical insomnia.

#### Rescue Drugs and Recurrence

A VAS score > 4 that occurred during postoperative follow-up was defined as recurrence of PHN in the study. For patients with relapse, the dosage of oral pregabalin was adjusted according to the specific situation.

#### Treatment Effectiveness

The assessment criteria (22) for pain relief was divided into five levels. Subjective symptoms and clinical signs were assessed at 6 months:

① Complete relief – 100% relief from pain
② Significant relief – pain relief degree ≥ 75%
③ Moderate relief – pain relief degree: 50–75%
④ Mild relief – pain relief is <50%
⑤ No effect – no relief at all

Total effective rate (%)=[(①+②+③)/n] ∗ 100%.

#### Postoperative Satisfaction

We evaluated patients' satisfaction with treatment outcomes with a five-point Likert scale, including very satisfied (5 points), satisfied (4 points), average (3 points), dissatisfied (2 points), and very dissatisfied (1 point).

#### Complications and Side Effects

These included puncture site bleeding, hematoma, hypertension, hypotension, bradycardia, pleural puncture, pneumothorax, lidocaine or MB adverse reactions, spinal cord injury, nerve injury, limb movement disorders, and sensory disorders. For every complication, we offered prompt treatment and recorded their progress in recovery.

### Statistical Analysis

Data were analyzed using SPSS version 19.0 statistical software (IBM, New York, United States). The measurement data were first tested for normality using the single-sample Kolmogorov-Smirnov test. Data following normal distribution were expressed as mean ± standard deviation (x ± SD). An independent sample *t*-test was used for comparison between groups. Changes in VAS, HAD, ISI, and satisfaction at each time point between groups were analyzed by repeated-measures ANOVA. Data not subject to normal distribution were expressed as median ± quartile spacing and variables were compared using the Kruskal–Wallis rank-sum test. The enumeration data were analyzed by chi-square test or Fisher's exact test; *p* < 0.05 was selected to represent statistical significance.

## Results

### Patient Characteristics

Preoperative general information, including sex, age, weight, height, BMI, pain duration, VAS, ISI, HAD, and daily dosage of pregabalin, did not differ significantly between the two groups (*p* < 0.05) (Table 1).

**Table 1:** Preoperative patient characteristics.

**Parameters**	**Group A**	**Group B**	***p*** **-value**
Patients (*n*)	36	36	–
Sex [F/M, (%)]	18 (50.0)/18 (50.0)	16 (44.4)/20 (55.6)	0.511
Age (years, range)	69.22 ± 6.97 (57–84)	71.55 ± 9.13 (51–89)	0.096
Height (cm, range)	166.27 ± 10.28 (150–194)	168.81 ± 9.09 (154–187)	0.568
Weight (kg)	66.09 ± 8.85	66.11 ± 7.54	0.423
BMI (kg/m^2)^	23.89 ± 2.29	23.20 ± 1.96	0.483
Presurgery pain duration (M, range)	7.86 ± 4.24 (3–20)	9.02 ± 5.71 (3–24)	0.104
Presurgery VAS	6.50 ± 0.74	6.50 ± 0.81	0.613
Presurgery ISI	21.31 ± 4.27	22.65 ± 4.06	0.189
Presurgery HAD	15.04 ± 3.09	15.92 ± 3.03	0.545
Pregabalin (mg/day)	380.21 ± 55.37	379.17 ± 57.94	0.884

*BMI, body mass index; F, female; M, male; M, median; VAS, Visual Analog Scale; HAD, Hospital Anxiety and Depression Scale; ISI, Insomnia Severity Index*.

*The results are expressed as mean ± standard deviation unless otherwise noted*.

### VAS Pain Scores

**Figure 3:**
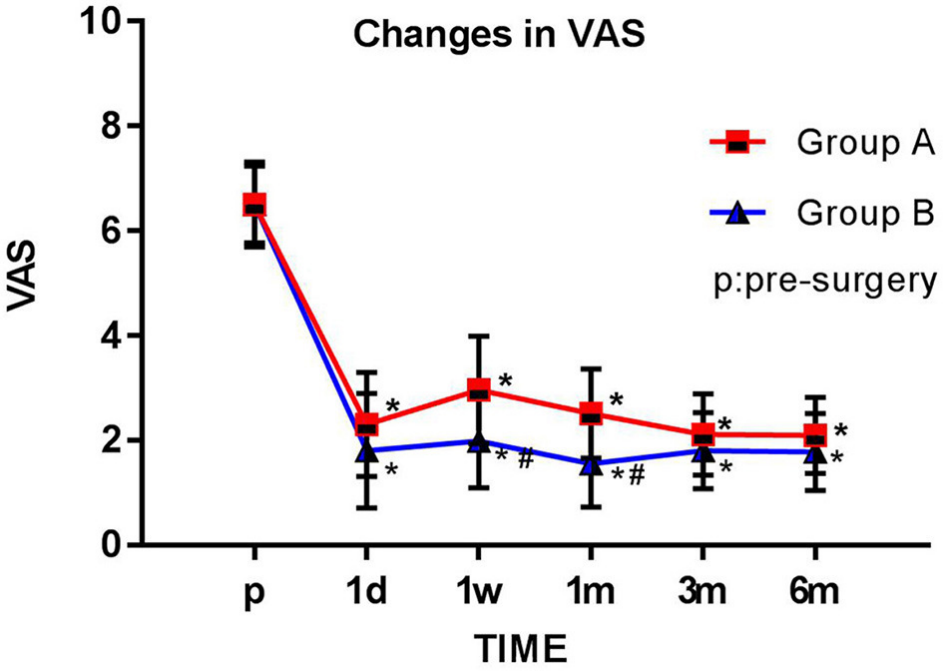
Comparison of preoperative and postoperative VAS scores. The Visual Analog Scale (VAS) score for each group is plotted for each time point examined. The results are expressed as mean ± standard deviation. *Compared with the presurgery score (p), *p* < 0.05; #compared with group A at the same time point, *p* < 0.05.

With treatment, VAS pain scores significantly lowered compared to presurgery values for all patients in both groups at each postsurgery observation time point (1 day, 1 week, 1 month, 3 months, and 6 months; all *p* < 0.05). At the 1-week and 1-month follow-up appointments, the VAS pain scores of group B were significantly lower than those of group A (*p* < 0.05) (Figure 3).

### Hospital Anxiety and Depression Scale

**Figure 4:**
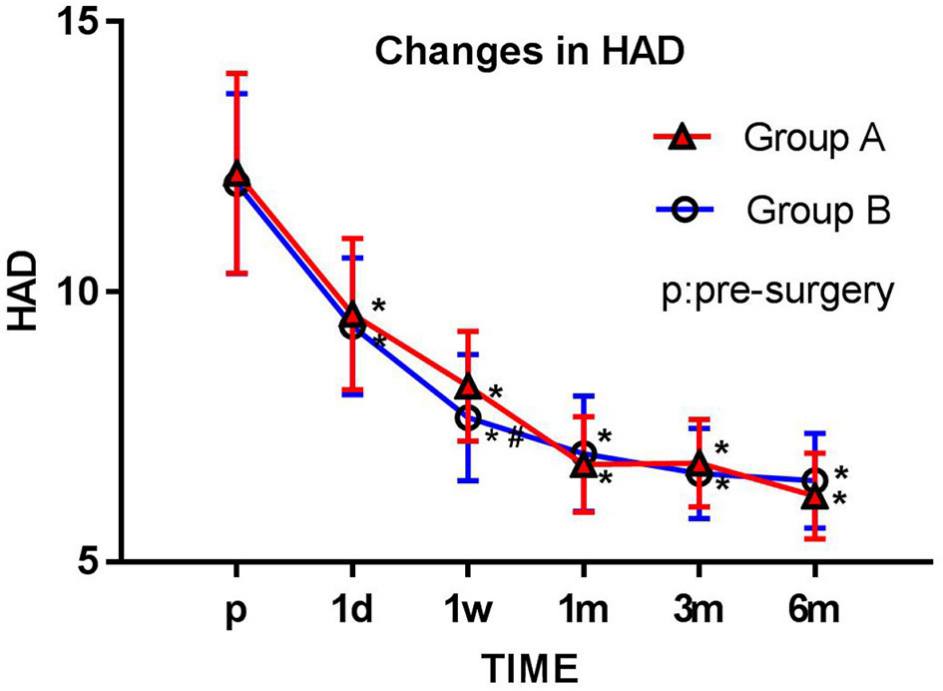
Comparison of preoperative and postoperative HAD scores. The Hospital Anxiety and Depression (HAD) score for each group is plotted for each time point examined. The results are expressed as mean ± standard deviation. *Compared with the presurgery score (p), *p* < 0.05; #compared with group A at the same time point, *p* < 0.05.

The HAD scores were significantly lower in both groups at each postsurgery observation time point (1 day, 1 week, 1 month, 3 months, and 6 months; all *p* < 0.05) than before surgery. The HAD scores in group B were significantly lower than those in group A 1 week after surgery (*p* < 0.05) (Figure 4).

### The Insomnia Severity Index

**Figure 5:**
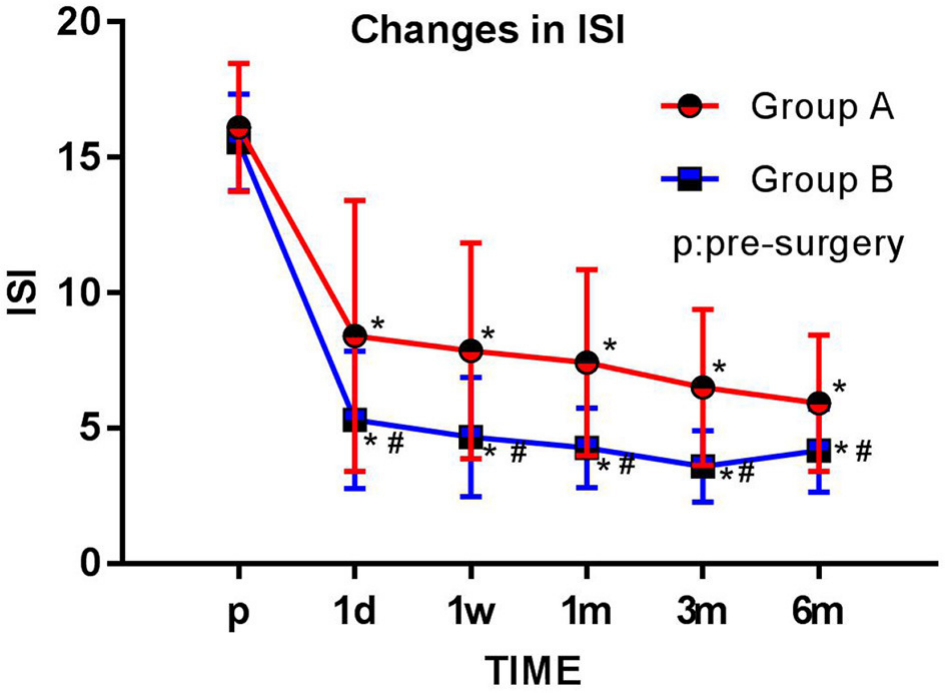
Comparison of preoperative and postoperative ISI scores. The Insomnia Severity Index (ISI) score for each group is plotted for each time point examined. The results are expressed as mean ± standard deviation. *Compared with the presurgery score (p), *p* < 0.05; #compared with group A at the same time point, *p* < 0.05.

The ISI scores were significantly lower in both groups at each postsurgery observation time point (1 day, 1 week, 1 month, 3 months, and 6 months; all *p* < 0.05) compared with preoperation values. The ISI scores of group B were significantly lower than those of group A at each observation time point (1 day, 1 week, 1 month, 3 months, and 6 months after surgery) (*p* < 0.05) (Figure 5).

### Dosage of Pregabalin

**Figure 6:**
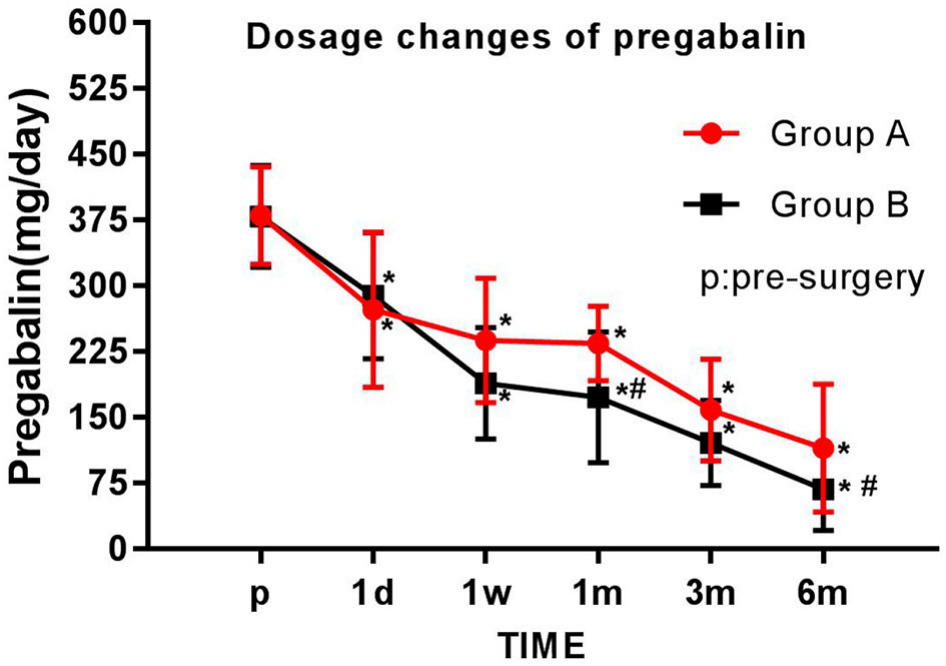
Comparison of pregabalin dosage before and after the operation. The daily pregabalin dosage taken by each group is plotted for each time point examined. The results are expressed as mean ± standard deviation. *Compared with the presurgery score (p), *p* < 0.05; #compared with group A at the same time point, *p* < 0.05.

The daily dosage of pregabalin was significantly lower at each postoperative time point (1 day, 1 week, 1 month, 3 months, and 6 months; all *p* < 0.05) than at preoperation. The dosage of pregabalin in group B was significantly lower than that in group A 1 and 6 months after surgery (*p* < 0.05) (Figure 6).

### Total Effective Rate

About 6 months after the operation, the total effective rate of group A and group B was 66.67 and 83.33%, respectively. The total effective rate of group B was significantly higher than that of group A (*p* < 0.05) (Table 2).

**Table 2:** Total effective rate in group A and group B.

	***n***	**Completely**	**Clearly**	**Moderately**	**Mildly**	**Ineffective**	**Total effective rate (%)**
Group A	36	1	4	19	10	2	66.67
Group B	36	2	10	18	5	1	83.33*

* *Compared with group A, p < 0.05*.

### Postoperative Satisfaction

A five-point Likert scale was used to assess patients' satisfaction with treatment outcomes. More than 80% of patients in each group were satisfied (4 points) or very satisfied (5 points) with the treatment. Patients in group B were significantly more satisfied at 1 week and 6 months after surgery than those in group A (*p* < 0.05) (Table 3).

**Table 3:** Patient satisfaction in group A and group B.

	**1 d**	**1 wk**	**1 mo**	**3 mo**	**6 mo**
Group A	3.96 ± 0.58	4.00 ± 0.61	3.94 ± 0.55	3.90 ± 0.56	3.09 ± 0.64
Group B	4.22 ± 0.66	4.42 ± 0.54^#^	4.21 ± 0.44	4.15 ± 0.50	4.21 ± 0.44^#^

*The results are expressed as mean ± standard deviation*.

# *Compared with group A, p < 0.05*.

### Adverse Reactions

No serious complications such as pneumothorax or intraspinal/paraspinal hematoma occurred in any of the patients. No cases withdrew from treatment due to adverse complications. Tachycardia, dizziness, nausea/vomiting, or hypertension occurred in three patients in group A and two patients in group B during PRF treatment, which were effectively alleviated after symptomatic treatment. After treatment, subcutaneous swelling at the puncture site appeared in one patient in group A and two patients in group B, which was relieved after a symptomatic cold compress. No significant differences were found in adverse reactions between group A and group B (*p* < 0.05) (Table 4).

**Table 4:** Complications in group A and group B (cases, %).

	**Tachycardia**	**Dizziness/ nausea/ vomiting**	**Hypertension**	**Subcutaneous swelling at the puncture**
Group A	1 (2.8)	1 (2.8)	1 (2.8)	1 (2.8)
Group B	0 (0.0)	0 (0.0)	2 (5.6)	2 (5.6)

## Discussion

The results of this study showed that patients treated with PRF + MB had more obvious pain relief, less oral analgesic drug dosage, less anxiety and depression, and significantly improved sleep, than those treated with PRF alone.

PRF utilizes high frequency and high voltage current pulses in the treatment area to cause slight temperature increases. Because the heat generated by the current can dissipate between pulses, the local temperature does not continue to rise. This allows PRF to ease PHN without changing the nerve fiber structure (23, 24). In this study, pain was relieved after PRF therapy, which is consistent with the findings of previous studies (10–13). However, other studies reported that PRF therapy efficacy in treating PHN was not ideal (14, 15). Ineffective PRF therapy in these cases may be due to a small number of cases, lax inclusion criteria, and the long duration of PHN (>5 years). One study showed that PRF combined with nerve block can effectively alleviate PHN (25). The results of the study presented here showed that the therapeutic effect of PRF combined with MB was superior to that of PRF alone. This is consistent with previous studies that have shown that MB efficacy can be maximized when used in combined treatment rather than as a monotherapy (26).

The possible mechanisms explaining how MB participates in pain relief are as follows: ① MB can stain unmyelinated nerve fibers, especially sensory nerve fibers, and promote neuroprotection by reducing cysteine activation and protecting mitochondrial membrane potential (17). ② MB also metabolizes glucose, promotes pyruvate oxidation, changes the acid-base balance and membrane potential inside and outside the nerve terminal membrane, impedes nerve impulse conduction, and produces an analgesic effect. ③ MB can cause reversible damage to the nerve to reduce nerve sensitivity without damaging the neuronal structure. In this manner, it can maintain local anesthesia for approximately 20 days and can be used as a nerve block to relieve pain, especially refractory neuropathic pain, for an extended time or permanently (16). ④ MB may promote nerve regeneration after injury by regulating the local microenvironment (26). In conclusion, MB can cause reversible damage to the nerve medulla making the nerve lose its sensitivity and achieving analgesic effect without damaging the structure of neurons.

In this study, CT was used for precise positioning of the therapy. This was advantageous because of the simple operation. It allowed for direct visualization of the puncture and drug dispersion, and it reduced the incidence of adverse puncture reactions, especially the incidence of pneumothorax. In addition, nerve sensory test and nerve motor test performed before PRF therapy increased the accuracy of locating nerves and further reduced the incidence of adverse puncture reactions.

Patients' sleep, emotional disorders, and satisfaction were evaluated using the ISI, HAD, and a five-point Likert scale, respectively. These evaluations have been adopted by many PHN studies (27, 28) and can accurately assess patients' status in a scientific and reasonable way. The results of this study showed that patients in group B had lower HAD scores than those in group A 1 week after surgery (*p* < 0.05), and group B's ISI scores were superior to group A's scores at 1 day, 1 week, 1 month, 3 months, and 6 months after surgery (*p* < 0.05). Patients in group B were more satisfied at 1 week and 6 months after surgery than those in group A (*p* < 0.05). Thus, this study shows that PRF combined with MB treatment is more beneficial than PRF alone for treating PHN as well as the effects PHN has on patients' sleep and mood.

In this study, <7% of patients experienced transient arrhythmias, dizziness, nausea/vomiting, or hypertension during PRF treatment, which were effectively resolved after symptomatic treatment. We believe that the occurrence of these adverse reactions was due to the patients' mental stress or adverse reactions to analgesics, and they were not caused by PRF or MB *per se*. After PRF treatment, one patient in group A and two patients in group B developed a transient local hematoma associated with the puncture process. No serious complications such as pneumothorax or intraspinal/paraspinal hematoma occurred during the operation. None of the patients treated with MB in this study had adverse allergic reactions or abnormal vital signs. However, six patients showed a change in their urine color, which returned to normal after 3 days.

This study has the following limitations: first, the sample size of our study is small, so it is necessary to increase the sample size and conduct a multi-center observation. Second, we did not perform subgroup analysis based on disease stage, age, or sex. We will continue to collect cases and refine subgroups with a goal of obtaining more in-depth results in the future. Finally, to be more relevant to clinical application, this study did not set the PRF-only treatment group and the MB-only treatment group.

In conclusion, CT-guided PRF combined with MB paravertebral nerve block is superior to PRF alone in the treatment of thoracic PHN. This method is safe, effective, can significantly relieve PHN, and can improve related sleep and emotional disorders. Clinicians should consider these results when choosing PHN treatment options.

## Data Availability Statement

The original contributions presented in the study are included in the article/supplementary material, further inquiries can be directed to the corresponding author/s.

## Ethics Statement

The studies involving human participants were reviewed and approved by Ethics Committee of Shengjing Hospital of China Medical University. The patients/participants provided their written informed consent to participate in this study. Written informed consent was obtained from the individual(s) for the publication of any potentially identifiable images or data included in this article.

## Author Contributions

MJ, PY, and ZH: conception and design of the study. MJ and DZ: acquisition of data. MJ and ZH: data analysis. MJ and PY: drafting the manuscript. All authors contributed to the article and approved the submitted version.

## Funding

This study was supported by the 345 Talent Project of Shengjing Hospital of China Medical University and the Livelihood Science and Technology Project of Liaoning Province (No: 2021JH2/10300040).

## Conflict of Interest

The authors declare that the research was conducted in the absence of any commercial or financial relationships that could be construed as a potential conflict of interest.

## Publisher's Note

All claims expressed in this article are solely those of the authors and do not necessarily represent those of their affiliated organizations, or those of the publisher, the editors and the reviewers. Any product that may be evaluated in this article, or claim that may be made by its manufacturer, is not guaranteed or endorsed by the publisher.
